# Social factors related to the clinical severity of influenza cases in Spain during the A (H1N1) 2009 virus pandemic

**DOI:** 10.1186/1471-2458-13-118

**Published:** 2013-02-07

**Authors:** José María Mayoral, Jordi Alonso, Olatz Garín, Zaida Herrador, Jenaro Astray, Maretva Baricot, Jesús Castilla, Rafael Cantón, Ady Castro, Miguel Delgado-Rodríguez, Alicia Ferri, Pere Godoy, Fernando Gónzález-Candelas, Vicente Martín, Tomás Pumarola, José María Quintana, Núria Soldevila, Sonia Tamames, Ángela Domínguez

**Affiliations:** 1 Servicio de Vigilancia epidemiológica de Andalucia, Sevilla, Spain; 2 Institut Municipal de Investigació Mèdica, Barcelona, Spain; 3 CIBER Epidemiología y Salud Pública, Barcelona, Spain; 4 Programa de Epidemiología Aplicada de Campo, ISCIII, Madrid, Spain; 5 Área de Epidemiología, Comunidad de Madrid, Madrid, Spain; 6 Instituto de Salud Pública de Navarra, Pamplona, Spain; 7 Hospital Universitario Ramón y Cajal, Madrid, Spain; 8 CIBER Enfermedades Respiratorias _Instituto de Investigación Hospital 12 de Octubre, Madrid, Spain; 9 Universidad de Jaén, Jaén, Spain; 10 Departament de Salut, Generalitat of Catalonia, Barcelona, Spain; 11 Centro Superior de Investigación en Salud Pública, Universitat de València, València, Spain; 12 Instituto de Biomedicina, Universidad de Leon, Leon, Spain; 13 Red Española de Investigación en Patología Infecciosa, Madrid, Spain; 14 Unidad de Investigación, Hospital Galdakao-Usansolo, San Sebastian, Spain; 15 Dirección General de Salud Pública e Investigación, Desarrollo e Innovación, Junta de Castilla y León, Valladolid, Spain; 16 Departament de Salut Pública, Universitat de Barcelona, Barcelona, Spain; 17 Sº de Epidemiología. Consejería de Salud. Andalucía, Avda. de la Innovación s/n, 41080, Sevilla, Spain

**Keywords:** Influenza A (H1N1) 2009, Pandemic, Hospitalization, Social factors

## Abstract

**Background:**

During the 2009 influenza pandemic, a change in the type of patients most often affected by influenza was observed. The objective of this study was to assess the role of individual and social determinants in hospitalizations due to influenza A (H1N1) 2009 infection.

**Methods:**

We studied hospitalized patients (cases) and outpatients (controls) with confirmed influenza A (H1N1) 2009 infection. A standardized questionnaire was used to collect data. Variables that might be related to the hospitalization of influenza cases were compared by estimation of the odds ratio (OR) and 95% confidence intervals (CI) and the variables entered into binomial logistic regression models.

**Results:**

Hospitalization due to pandemic A (H1N1) 2009 influenza virus infections was associated with non-Caucasian ethnicity (OR: 2.18, 95% CI 1.17 − 4.08), overcrowding (OR: 2.84, 95% CI 1.20 − 6.72), comorbidity and the lack of previous preventive information (OR: 2.69, 95% CI: 1.50 − 4.83). Secondary or higher education was associated with a lower risk of hospitalization (OR 0.56, 95% CI: 0.36 − 0.87)

**Conclusions:**

In addition to individual factors such as comorbidity, other factors such as educational level, ethnicity or overcrowding were associated with hospitalization due to A (H1N1) 2009 influenza virus infections.

## Background

The pathogenesis of infectious diseases is generally well known. Biomedical advances have resulted in a wide understanding of the most common infectious agents. However, individual and social factors also affect the morbidity and mortality of infectious diseases and may not be as easy to identify, measure or modify. These factors also seem to contribute to the differences in disease incidence, evolution and severity detected between individuals and population groups [1-7].

Objective material conditions and available individual and social resources influence the health status, regardless of the health problems presented [8,9]. This relationship has been widely studied, especially in the context of chronic disease. However, applying this approach to acute infectious diseases may be more difficult, for reasons which include the short period of disease incubation and duration and accessible, efficacious treatments [10,11].

After the A (H1N1) 2009 influenza virus pandemic, some studies have analyzed the role of specific clinical variables as risk factors for the development of severe influenza. However, studies on the 1918 pandemic point to overcrowding and hunger as the main causes of the rapid expansion of cases throughout Europe [12]. Although individual factors such as nutrition, and the socioeconomic or educational level are less-frequently considered when studying influenza, there is sufficient evidence of their impact on health and disease evolution to warrant study of their possible role in severe cases of influenza [13-16].

This is especially relevant in the case of A (H1N1) 2009 influenza virus infections, given the profile of severe cases reported during the pandemic. A major epidemiological difference between pandemic influenza virus A (H1N1) 2009 infections and previous seasonal influenza infections was that the greatest proportion of severe cases occurred in young people and young adults, resulting in an increase in the potential life years lost [17-22]. However, the virulence of the disease was particularly high among people aged ≥ 65 years: while the incidence in the community was low in this age group, the proportion of severe, hospitalized cases was higher. In Spain, it is estimated that 2.5% of all cases of influenza attended by primary care centres were in people aged ≥ 65 years, compared with 10.6% admitted to hospital intensive care units [23].

Other determinants, such as differences in access to preventive pharmacological and non-pharmacological measures have also been show to play a role in infections of acute onset [16,24]. Study of access to and the use of health services may be valuable not only with respect to the clinical evolution of cases but also in determining how well they function in an atypical situation such as the 2009 pandemic [25-28]. Few studies of severe cases of influenza have included non-clinical factors even though it is known that vulnerability to infectious diseases is mediated by individual and social conditions and that these are a European public health priority [1,29]. The aim of this study was to analyze individual and social factors associated with hospitalization during the influenza A (H1N1) 2009 pandemic.

## Methods

### Study design

We performed a multicenter matched case–control study. Patients were recruited from hospitals and primary healthcare clinics and emergency units in seven Spanish regions (Andalusia, Catalonia, Castile and Leon, Madrid, Navarre, Basque Country and Valencia Community) between July 2009 and February 2010, during the peak of the influenza A (H1N1) 2009 pandemic in Spain [24].

The sample size required to detect a relative risk (OR) of 1.5 assuming a prevalence of the investigated factors in outpatients of 0.15, a bilateral level of significance of α = 0.05 and a power of β = 0.80 was 654, using the criteria proposed by Schlesselman [30].

Selection of cases and controls:

Hospitalized cases were defined as all patients admitted during the study period due to influenza-like illness, respiratory infection (including pneumonia), septic shock or multiple organ failure in whom pandemic influenza virus A (H1N1) 2009 infection was confirmed. Nosocomial cases were excluded.

For each hospitalized case, one control was identified among patients attended by primary health care clinics and emergency units due to influenza-like illness or respiratory infection in whom A (H1N1) 2009 infection was confirmed using the same diagnostic techniques as for cases. Controls were matched with cases according to age (±6 months in patients aged 0–2 years; ±3 years in patients aged 3–17 years and ±5 years in patients aged > 17 years), date of hospitalization of the case (±21 days) and province of residence of the case. If there was more than one exact match, the control selected was the first patient attended in an outpatient centre from the same province as the hospitalized case whose sample was laboratory confirmed.

Exclusion criteria for cases and controls were failure to answer the questionnaire and refusal of informed consent.

Influenza virus A (H1N1) 2009 infection was confirmed in cases and controls by real time RT-PCR. After giving informed consent to participate in the study, cases and controls were administered a structured questionnaire by specifically-trained health professionals in order to collect sociodemographic and medical information on unfavourable medical factors (smoking, morbid obesity [body mass index (BMI) ≥ 40], hypertension, lung disease, cardiovascular disease, kidney failure, diabetes, chronic liver disease, immunodeficiency, disabling neurological disease, malignancy, transplantation, cognitive dysfunction, seizure disorders and rheumatic diseases) and information on preventive measures received through various means (health centres, the media, internet, etc.) during the previous year on preventing the spread of influenza and antecedent of influenza pandemic vaccination. The social variables of interest in this study were the educational level, overcrowding (below the fifth percentile of the distribution of square metres available per person in the normal residence of all study participants), ethnicity and the use of outpatient healthcare services, family physicians or non-hospital emergency units due to influenza symptoms in the 7 days before hospital admission in cases and a medical visit due to influenza symptoms in controls.

Cases and controls were offered the possibility of completing the survey in person at a health centre or by telephone.

The median time from hospitalization of cases or taking the sample from outpatient controls until the survey was calculated.

### Statistical analysis

A descriptive analysis was made of the characteristics of cases and controls. A bivariate analysis was made using McNemar’s *χ*2 test for matched categorical variables. A multivariate analysis was performed using binomial logistic regression which included all variables of interest and possible confounding factors. Associations were measured using crude and adjusted odds ratios (OR) and their 95% confidence intervals (CI).

The multivariate models were calculated using Cox conditional logistic regression models.

Variables were selected manually and entered in an initial model that included all selected variables. Variables of interest and significant variables in the bivariate analysis were entered in the model. The probability of the steps for entry was 0.05 and the output 0.10. The cutoff point for iteration was 0.5 and the maximum number of iterations was 20. To assess whether this was an appropriate model, the Wald statistic was calculated. The criterion for selecting a new model was determined by the coefficient of likelihood of the model. The analysis was made using the SPSS v13.0 statistical package.

The variable age was included as a continuous variable in order to adjust the multivariate model.

#### Data confidentiality and ethical considerations

All information collected was treated as confidential according to the law on observational studies. The study was approved by the Ethics Committee of the hospitals involved (Clinical Research Ethics Committee, Hospital Costa del Sol; Autonomous Clinical Trials Committee of Andalusia; Clinical Research Ethics Committee, Clinica de Leon; Clinical Research Ethics Committee, Municipal Institute of Healthcare (CEIC-IMAS); Clinical Research Ethics Committee, Corporación Sanitaria ParcTaulí of Sabadell; Research Committee, Sant Joan de Déu University Hospital; Clinical Research Ethics Committee, Basque Country; Clinical Research Ethics Committee, Doctor Peset Univeristy Hospital, Valencia; and, Clinical Research Ethics Committee, Clinical Research Ethics Committee, General Directorate of Public Health, Valencia.

Information was collected after written informed consent was obtained from all patients.

## Results

A total of 704 possible eligible hospitalized cases and 716 possible eligible controls were considered for the study. Five hospitalized cases and eleven controls were excluded because they did not give consent to participate and 6 possible eligible controls were excluded because failure to response the questionnaire. So, finally a total of 699 cases and 699 controls met the inclusion criteria and were included in the analysis. Baseline characteristics of cases and controls are shown in Table 1.

**Table 1:** Characteristics of matched cases (hospitalized inpatients) and controls (outpatients) with confirmed pandemic influenza A (H1N1) 2009

**Characteristic**		**Cases**	**Controls**
		**N (699)**	**N (699)**
Mean age (*)		37.82	33.69
Sex	Male (*)	342 (49.1)	301 (43.1)
	Female (*)	356 (50.9)	397 (56.9)
Ethnicity	White (**)	588 (87.1)	634 (94.1)
	Roma (**)	15 (2.2)	2 (0.3)
	Amerindian (**)	46 (6.8)	16 (2.4)
	Arab or North African (*)	17 (2.5)	6 (0.9)
	Other	10 (1.5)	18 (2.7)
Educational level	No formal education (**)	50 (7.7)	24 (3.7)
	Primary (**)	213 (32.8)	128 (19.7)
	Secondary	295 (45.4)	311 (47.8)
	Higher (**)	92 (14.2)	187 (28.8)
Overcrowding	> = 29 m^2^/p	234 (45.7)	252 (49.2)
	28 - 15 m^2^/p	237 (46.3)	240 (47.1)
	<= 14 m^2^/p (**)	41 (8)	20 (3.7)
Unfavourable medical factors	0 (**)	242 (34.6)	382 (54.6)
	1	230 (32.9)	238 (34)
	2 (**)	122 (17.5)	58 (8.3)
	>2 (**)	105 (15)	21 (3)
Prior preventive information	Yes (**)	577 (82.5)	639 (91.4)
	No (**)	122 (17.5)	60 (8.6)
Prior pandemic vaccination	No	691 (99)	697 (99.9)
	Yes	7 (1)	1 (0.1)
Previous outpatient care	No (**)	357 (51.1)	557 (79.7)
	Yes (**)	342 (48.9)	142 (20.3)
Previous emergency care	No (**)	488 (69.8)	603 (86.3)
	Yes (**)	211 (30.2)	96 (13.7)

(*) P <0.05, (**) p <0.01.

The median time from hospitalization of cases or taking the sample from outpatient controls until the survey was 99 days (range 0 to 404) for hospitalized cases and 155 days (range 0 to 414) for outpatient controls.

No significant differences were found between sexes in the proportion of hospitalized cases. A total of 87.1% of cases and 94.1% of controls were Caucasian, 2.2% of cases and 0.3% of controls were Roma (p = 0.002), 6.8% of cases and 2.4% of controls were Amerindian (p <0.001) and 2.5% of cases and 0.9% of controls were Arab or North African (p <0.05).

Significantly more cases had no (7.7%) or primary education (32.8%) than controls (3.7% and 19.7% respectively, p <0.001). In contrast 14.2% of cases and 28.8% of controls had a higher education (p <0.001).

Significantly more cases (8.0%) than controls (3.7%) had a living space of less than 14 m^2^. Significantly fewer cases (34.6%) than controls (54.6%) had no risk factor or comorbidity (p <0.001). In contrast, more cases than controls had ≥ 2 risk factor (p < 0.001). Likewise, 17.5% of cases but only 8.6% of controls had received no information about preventing the transmission of influenza. Visits to primary care or hospital emergency services in the days before infection were significantly higher in cases than in controls (p <0.001).

Table 2 shows the results of the bivariate and multivariate analyses. No significant differences were found between sexes in the proportion of hospitalized cases. Hospitalization was associated with non-Caucasian ethnicity (Roma, Amerindian, Arab or North African) (OR 2.18, CI (95%): 1.17 − 4.08), and personal space < 14 m^2^ per person (OR: 2.84, 95% CI 1.20 − 6.72). Patients with secondary or higher education had a lower probability of hospitalization than patients without formal education or with only primary education (OR: 0.56; 95% CI: 0.36-0.87).

**Table 2:** Comparison of cases (hospitalized patients) and controls (outpatients) with confirmed pandemic influenza A (H1N1) 2009

**Independent variables**		**Cases(N = 699)**	**Controls (N = 699)**	**Crude OR (95%)**	**Adjusted OR (95%)**
Sex	Male	342	301	1	1
	Female	356	397	0.79 (0.61 – 1.01)	0.72 (0.51 – 1.01)
Ethnicity	White	588	634	1	1
	Other	88	42	2.39** (1.45 – 3.98)	2.18** (1.17 – 4.08)
Educational level	No/primary education	263	152	1	1
	Secondary or higher	387	498	0.38** (0.27 – 0.53)	0.56** (0.36 – 0.87)
Overcrowding	> = 29 m^2^	234	252	1	1
	28 – 15 m^2^	237	240	1.07 (0.78 – 1.49)	1.09 (0.76 – 1.57)
	<= 14 m^2^	41	20	3* (1.04 – 10.55)	2.84* (1.20 – 6.72)
Unfavourable medical factors	0	242	382	1	1
	1	230	238	1.78** (1.25 – 2.53)	1.60* (1.07 – 2.40)
	2	122	58	3.99** (1.99 – 8.03)	2.94** (1.61 – 5.36)
	>2	105	21	38** (6.42 – 153.99)	18.51** (6.08 – 56.41)
Previous preventive information	Yes	577	639	1	1
	No	122	60	2.38** (1.56 – 3.66)	2.69** (1.50 – 4.83)
Prior pandemic vaccination	No	691	697	1	1
	Yes	7	1	–	–
Previous outpatient care	No	357	557	1	1
	Yes	342	142	4.57** (3.23 – 6.51)	3.46** (2.22 – 5.39)
Previous emergency care	No	488	603	1	1
	Yes	211	96	3.02** (2.10 – 4.35)	2.68** (1.66 – 4.32)

Results of the bivariate analysis (crude OR) and conditional logistic regression (adjusted OR).

(*)P <0.05, (**) p <0.01.

Receiving information on preventive measures was a protective factor against hospitalization. The probability of hospitalization was higher in patients who had received no information (OR: 2.69 95% CI: 1.50 − 4.83).

Hospitalization due to influenza increased according to the number of risk factors and comorbidities (OR: 1.60, 95% CI: 1.07 − 2.40 for one, OR: 2. 94, 95% CI 1.61 − 5.36 for two and OR: 18.51, 95% CI 6.08 − 56.41 for ≥3 risk factors or comorbidities).

During the pandemic period, hospitalized cases made more visits to primary care centres (OR 3.46, 95% CI 2.22 − 5.39) and outpatient emergency services (OR 2.68, 95% CI 1.66 − 4.32) compared with controls.

## Discussion

The relationship between specific unfavourable medical conditions and the severity of influenza infection has been widely studied with respect to the 2009 pandemic [23,31,32]. The number of comorbidities, which increases with age, was an important factor in this respect, as shown by other studies made of the pandemic [23,31]. The World Health Organization working group on risk factors associated with severe A (H1N1) 2009 virus infection found that the proportion of cases with chronic disease increased significantly in tandem with disease severity. The risk factors were similar to those of seasonal influenza, except for differences related to age [32].

Spanish studies have found a greater frequency of comorbidity in people aged ≥ 65 years, which was associated with hospitalization [20,23]. Our results also show that the probability of hospitalization increased in tandem with the number of comorbidities, and that the influence of these predisposing factors was independent of other factors analysed.

Various studies have shown a relationship between the health status and social inequalities [2-7] that goes beyond access to health services to include other factors such as income, housing, education, ethnicity, etc., which may determine the gradient of health between social groups [33,34]. The present study assessed some of these factors, including the educational level, ethnicity, overcrowding in living conditions, and access to preventive information and primary care services.

Given that influenza is transmitted aerially, overcrowding or workplace characteristics [35] or the use of public transport [36] can also predispose to a greater risk of exposure. Like other studies, we found a significant association between disease severity and a greater degree of overcrowding, even with a threshold for overcrowding that may be considered moderate [37]. Although we did not find that this effect was modified by other factors such as ethnicity or educational level, we suggest that this association may be due to the relationship between overcrowding and income levels, which influence disease severity.

Spanish [38] and other [39,40] descriptive studies show differences in the use of health services and in hospitalization due to the complications of influenza during the 2009 pandemic in immigrants and ethnic minorities, on the one hand, and indigenous people on the other hand. We found that non-Caucasian ethnicity was associated with greater disease severity. Although the small number of non-Caucasians included in the study did not permit analysis by ethnic groups in the multivariate models. However, the descriptive statistics suggest that greater disease severity occurred especially in Roma and North African or South American immigrants.

Studies have shown that preventive measures, including influenza vaccination, reduce mortality due to pneumonia and influenza in socially-deprived areas [16]. The fact that more-highly educated people have a higher socioeconomic level, have more information on avoiding risks and more-easily access health services [41] for general health care and for early care of other underlying conditions that may worsen the severity of influenza, may explain the association we found between lower educational levels and disease severity.

We found that hospitalized patients more-frequently attended primary care clinics and emergency units in the days before the onset of influenza. This may be related to the previous health status of hospitalized patients. Because the Spanish health system has universal coverage, it seems unlikely that inequalities in access to health care, as suggested by other authors [42], could explain our results. Although the study design did not permit assessment of the degree of compliance with preventive measures, people who had received preventive information had fewer hospitalizations.

This study has some limitations. The control group was not representative of the whole population infected, as it was selected from patients who visited their family physician for treatment for influenza. However, we believe this does not invalidate the results with respect to the study objectives. Both cases and controls were asked if they had received previous preventive information: however, we do not know to what degree this information was followed. However, given that we found significant differences in the results between patients who did and did not receive preventive information, the differences may have been even greater in those patients who did follow the advice given. Likewise, we were unable to evaluate the effect of previous vaccination or early antiviral treatment due to the small number of study patients who received these measures within the time required to evaluate them, even though vaccination was included in the adjusted analysis. The large number of missing values found for the variable overcrowding was assessed by comparing the characteristics of pairs with and without information on this variable. We found no significant differences, suggesting that the missing values do not compromise the validity of the results. However, other social and environmental factors of interest may not have been collected and analysed and therefore some residual confounding cannot be ruled out.

Efforts to reduce the impact of social inequality on the effects of communicable disease epidemics, such as influenza, must be undertaken not only by front-line health care services but also by preventive services and non-health care agents. Health interventions can reduce, but not eliminate health inequalities, as other sociodemographic determinants are not susceptible to these interventions and require a multidisciplinary approach. The adoption of plans and interventions that address the differential demographic impact of influenza may help to partially prevent the consequence of social inequalities on disease outcomes [1,11]. However, awareness of the risk of the “inverse care law” [43], that is, increased inequality due to the higher social classes having better access to preventive interventions should also be heightened.

## Conclusions

The main conclusions of this study are that, in addition to individual factors such as pre-existing unfavourable medical conditions, other social factors, such as non-Caucasian ethnicity, the lack of information on preventive measures, overcrowding, and a primary education or less, increased the risk of hospitalization due to confirmed influenza.

## Competing interests

All the authors declare that no financial competing interest exists with any companies or organizations whose products or services may be discussed in this article and that there are no non-financial competing interests (political, personal, religious, academic, ideological, intellectual, commercial or any other) in relation to the manuscript.

## Authors’ contributions

JMM directed the study and drew up the manuscript, together with ZH; AF and JMM carried out the statistical analysis; AD, OG and JA supervised the statistical analysis and the drawing up of the manuscript. JMM, AD and AF had full access to all the study data and take responsibility for the data and the accuracy of the analysis; NS and MB, supervised the administration of the data base; JA, RC, JC, AC, MDR, PG, FGC, VM, TP, JMQ and ST contributed to the design and supervision of the study and critical analysis of the article. The members of the CIBERESP Cases and Controls in Pandemic Influenza Working Group designed the case–control study on which this article is based. The other members of the CIBERESP Cases and Controls in Pandemic Influenza Working Group contributed to patient recruitment, data collection and interpreting the study results and have given final approval of the version to be published.

## Pre-publication history

The pre-publication history for this paper can be accessed here:

http://www.biomedcentral.com/1471-2458/13/118/prepub
